# Braving Difficult Choices Alone: Children's and Adolescents' Medical Decision Making

**DOI:** 10.1371/journal.pone.0103287

**Published:** 2014-08-01

**Authors:** Azzurra Ruggeri, Michaela Gummerum, Yaniv Hanoch

**Affiliations:** 1 Max Planck Institute for Human Development, Berlin, Germany; 2 University of Plymouth, Plymouth, England; University of Missouri-Kansas City, United States of America

## Abstract

**Objective:**

What role should minors play in making medical decisions? The authors examined children's and adolescents' desire to be involved in serious medical decisions and the emotional consequences associated with them.

**Methods:**

Sixty-three children and 76 adolescents were presented with a cover story about a difficult medical choice. Participants were tested in one of four conditions: (1) own informed choice; (2) informed parents' choice to amputate; (3) informed parents' choice to continue a treatment; and (4) uninformed parents' choice to amputate. In a questionnaire, participants were asked about their choices, preference for autonomy, confidence, and emotional reactions when faced with a difficult hypothetical medical choice.

**Results:**

Children and adolescents made different choices and participants, especially adolescents, preferred to make the difficult choice themselves, rather than having a parent make it. Children expressed fewer negative emotions than adolescents. Providing information about the alternatives did not affect participants' responses.

**Conclusions:**

Minors, especially adolescents, want to be responsible for their own medical decisions, even when the choice is a difficult one. For the adolescents, results suggest that the decision to be made, instead of the agent making the decision, is the main element influencing their emotional responses and decision confidence. For children, results suggest that they might be less able than adolescents to project how they would feel. The results, overall, draw attention to the need to further investigate how we can better involve minors in the medical decision-making process.

## Introduction

As part of an attempt to increase children's participation in decision making, Articles 12 and 13 of the United Nations Convention on the Rights of the Child specify that minors have the right to express themselves freely, be heard on all matters affecting them, and have their views taken seriously [1]. In recent years, there has been a shift from a paternalistic medical model, where physicians and parents hold an authoritative role in determining a child's treatment, to one advocating minors' involvement in their medical treatment [2]. Simultaneously, the US Supreme Court has come to recognize that minors who show maturity and competence deserve a voice in determining their medical treatment and even allows minors, in cases such as abortion, treatments for substance abuse and sexually transmitted diseases, and contraception, to receive treatment without parental consent or notification [3]. According to the Article 6 of the Convention for the Protection of Human Rights and Dignity of the Human Being with regard to the Application of Biology and Medicine: Convention on Human Rights and Biomedicine, ratified in Italy in 2001, “the opinion of the minor shall be taken into consideration as an increasingly determining factor in proportion to his or her age and degree of maturity.” Yet, a number of important questions remain open. Do children and adolescents welcome this change, wishing to be actively involved and taking responsibility for medical decisions regardless of the severity of the decision? Can they anticipate their emotional reactions to these choices?

Research on shared medical decision making among minors has so far focused on legal and ethical issues (e.g.,[4]), cognitive competency (e.g., [5]), and providing recommendations for determining children's level of involvement [2]. Although these are important issues, researchers have neglected to examine minors' views and feelings about this decision-making process. To the best of our knowledge, this is the first study to investigate (a) children's and adolescents' desire for autonomy, (b) their confidence that the right decision was made, and (c) their emotional reactions when faced with what Botti, Orfali, and Iyengar [6] called “tragic” medical choices.

What did Botti et al. mean by tragic choices? Imagine facing the following scenario: A premature baby's life is sustained by a ventilator, and after 3 weeks of treatment the baby's condition has not improved. The attending physician informs you (the parent) that you have a choice between continuing the treatment (with 40% probability of death or a crippling neurological condition if the baby survives) or withdrawing the treatment (resulting in the baby's death). Moreover, envision that you can make the decision yourself or have the physician assume responsibility for the decision [6]. Thus, according to Botti et al., tragic choices are ones that are difficult or distressing to make and have no clear positive outcome for the decision maker.

In three studies, Botti et al. [6] examined adults' desire for autonomy and their emotional reactions to this and other hypothetical dilemmas. They showed that adults for whom the doctor made the decision reported significantly fewer negative emotions than adults who made the choice themselves. They proposed that ascribing personal causation to an event intensifies negative emotions associated with a difficult choice. Consequently, it is possible that “individuals are likely to be better off if those choices are either physically or psychologically removed from them [6]. Two additional findings from Botti et al. study are of interest. First, not informing participants about treatment options and their outcomes eliminated the emotional advantages associated with transferring the choice to another agent. Second, despite feeling worse after the decision, choosers were reluctant to give up their autonomy.

Whether children and adolescents behave and react similarly when making difficult choices is an open and important question. If medical professionals are to include minors in the medical decision-making process, there may be times when they have to present minors with difficult choices (e.g., treatment options for diabetes, see [7]). In this study, we first manipulated who made the decision: the minor or the minor's parents; second, we manipulated which option was chosen by parents; and, finally, we manipulated whether information was given about all the possible treatment options. This allowed us to examine which of these factors (agent making the choice, choice taken, information provided) affect children's and adolescents' decision confidence and emotional reactions in difficult choice situations and, ultimately, whether minors prefer to make a difficult choice themselves, despite being able to anticipate the negative emotional consequences associated with this choice. Given the paucity of data on the topic, our investigation could have clinical implications for physicians (and possibly parents) who must decide whether to include minors in the medical decision-making process.

There is good evidence that adolescents in particular are increasingly interested in making decisions independent of adults [8], [9]. Compared to children, adolescents regard more issues as a matter of personal choice, have a stronger desire to be independent, and are more likely to question authority figures' decisions [10]. Hence, we expected that when faced with difficult medical decisions, adolescents (compared to children) would show a stronger preference for making autonomous decisions (Hypothesis 1).

According to Botti et al. [6], being responsible of a decision intensifies negative emotions associated with a difficult choice. Thus, despite children's and adolescents' willingness to make a decision autonomously, we would expect participants to experience a less negative emotional response when the difficult choice was made by their parents (Hypothesis 2). Indeed, even though adolescents want to be autonomous decision-makers, they are still seeking advice from a person they consider more competent and knowledgeable than themselves [11]. This is particularly true when decisions involve physical harm or moral and social-conventional transgressions [12]. Adolescents acknowledge that authority-based decision procedures can be more suitable in some environments where adults might have more competence and better knowledge (e.g., in school;[13], [14]).

Similarly, the confidence that the best decision was made might depend on either the agent (minor or parent) making the decision or the decision option chosen. We therefore explored two alternative—but not mutually exclusive—hypotheses: (a) If the agent making the decision is the most important element, we expected that participants, and especially adolescents, would show higher decision confidence when they made the decision themselves than when the parents made the decision, independent of the decision option chosen by the parents; (b) if the decision option chosen influences decision confidence, participants should be equally confident that the right decision was made when one particular option was chosen, independent of who (they themselves or the parents) made the decision (Hypotheses 3a and 3b respectively).

We also investigated *which* decision children and adolescents take, and explored whether and how the decision taken affected desire for autonomy, emotional response and decision confidence.

Finally, we hypothesized that minors would be sensitive to the information provided to make a decision (Hypothesis 4). That is, the negative emotional response and the confidence that the best decision was made would be worse if no information about the treatment options and outcomes were provided (see [6]).

## Methods

### Participants

Sixty-three 4th-grade children, aged 8 to 11 years (29 female, *M*_age_ = 9.6 years, SD = 0.6), and 76 high school students, aged 15 to 17 years (43 female, *M*_age_ = 16.5 years, SD = 0.7), were recruited from two schools in Livorno, Italy. Participants were all white, and none of them suffered a chronic medical or psychiatric condition that could have constrained or influenced a correct and neutral understanding of the instructions.

The experimental procedures were approved by the ethics committee of the Max Planck Institute for Human Development, and all the parents of the children involved, as well as the teachers and the schools' Institutional Review Board, were informed and consented (in written form) to let the children participate prior to data collection. Participants were asked to give their assent to participate, and were free to leave the classroom and withdraw from the experiment at any time.

### Design and procedure

All participants received a piece of paper with an introduction to a scenario, common to all conditions, and a description of the specific condition to which they were assigned (see below). All participants of one age group assigned to the same condition were tested together. The experimenter read the scenario aloud. Participants were asked to imagine they had had an accident 1 week before: They had been hit by a car while walking home from school. As a result of the accident, they had a broken leg and were suffering from a severe infection. After the first 10 days of treatment they did not get any better.

Participants were randomly assigned to one of four conditions: 1) own informed choice; 2) informed parents' choice to amputate; 3) informed parents' choice to continue the treatment; and 4) uninformed parents' choice to amputate. The number of participants in each condition for each age group is presented in Table 1. In the informed Conditions 1, 2, and 3, the doctor presented to the participants and their parents two alternatives: Continue the treatment or amputate the leg. If they continued with the treatment, there were 4 chances out of 10 that the infection would dangerously spread, and 6 chances out of 10 that the doctors would save the leg. Even if the doctors saved the leg, it would be seriously damaged and would hurt a lot, and the participant would not be able to run again. These survival odds were the same as in the Botti et al. [6] study but presented in a frequency format to make them easier for children to understand [15].

**Table 1:** Means and Standard Deviations for Participants' Preference for the Condition They Were Assigned to^a^, Choice Made in Condition 1, Willingness to Have Been Assigned to the Other Type of Choice Condition^a^^,^ b, Emotional Response^c^, and Decision Confidence^a^.

	Children		Adolescents		Total	
	*M*	SD	*M*	SD	*M*	SD
Condition 1: Own informed choice (amputate)						
Prefer my choice condition	5.9	3.4	7.0	1.4	6.1	3.0
Prefer the other type of choice condition	3.3	2.8	2.5	2.1	3.1	2.6
Emotional response	4.2	1.8	4.8	1.4	4.3	1.7
Decision confidence	6.4	3.6	6.0	0.0	6.3	3.2
Condition 1: Own informed choice (not amputate)						
Prefer my choice condition	8.3	1.2	7.0	2.0	7.2	1.9
Prefer the other type of choice condition	2.7	2.9	1.9	1.2	2.0	1.5
Emotional response	4.7	1.0	6.3	1.5	6.0	1.5
Decision confidence	8.7	0.6	7.3	1.9	7.5	1.8
Condition 2: Informed parents' choice (amputate)						
Prefer my choice condition	3.4	1.8	2.1	1.7	2.5	1.8
Prefer the other type of choice condition	6.4	2.8	7.9	1.3	7.4	2
Emotional response	6.6	1.3	7.9	0.6	7.5	1.0
Decision confidence	2.2	1.8	4.8	2.0	3.9	2.3
Condition 3: Informed parents' choice (not amputate)						
Prefer my choice condition	3.6	2.8	3.0	2.4	3.3	2.6
Prefer the other type of choice condition	5.0	3.5	7.6	1.7	6.1	3.2
Emotional response	4.6	2.1	5.6	1.7	5.0	2.0
Decision confidence	6.2	3.1	7.1	1.8	6.6	2.7
Condition 4: Uninformed parents' choice (amputate)						
Prefer my choice condition	3.3	3.2	2.5	1.9	2.8	2.4
Prefer the other type of choice condition	6.5	3.3	7.6	1.6	7.3	2.3
Emotional response	6.0	1.3	7.5	0.9	7.0	1.3
Decision confidence	3.3	2.6	4.7	2.5	4.3	2.6

a On a scale from 1, *not at all*, to 9, *extremely*.

b For participants who made their own choice (Condition 1), switching to parents' choice (Condition 2, 3 and 4) and vice versa.

c Average of five negative emotions (nervous, upset, unhappy, concerned, guilty), each reported on a scale from 1, *not at all*, to 9, *extremely*.

In Condition 1 (own informed choice), participants were asked to decide whether to amputate their leg. In Condition 2 (informed parents' choice), participants' parents made the decision to amputate the leg. In Condition 3 (informed parents' opposite choice), participants' parents made the decision not to amputate but to continue the treatment. In Condition 4 (uninformed parents' choice), the doctor did not mention the option to continue the treatment nor the outcome probabilities associated with the two alternatives, and the decision to amputate the leg was made by the parents.

After hearing the scenario, each group completed a questionnaire, almost identical to the one administrated by Botti et al. [6]. Only participants in Condition 1 (own informed choice) were asked for their decision on whether to amputate the leg and their reasons for their choice. Participants in all conditions were asked to indicate to what extent each of five negative emotions (nervous, upset, unhappy, concerned, guilty) described how they felt about the treatment decision on a scale from 1, *not at all*, to 9, *extremely*. (We left out one of the emotional states from the original questionnaire, “distressed,” as in Italian the two words for “distressed” and “concerned” are hard to tell apart). Next, participants had to indicate how confident they were that the best decision had been made on a scale from 1, *not at all*, to 9, *extremely*. The final two questions measured participants' preference for decision autonomy. Participants in Condition 1 (own informed choice) were asked how much they liked having to make the decision and how much they would have preferred that their parents made the decision for them. Participants in Conditions 2, 3, and 4 (informed parents' choice, informed parents' opposite choice, uninformed parents' choice) were asked how much they liked not having to make the decision and how much they would rather have made the choice themselves. The response scale for both questions ranged from 1 (*not at all*) to 9 (*extremely*).

A similar scenario and questionnaire was piloted with 128 participants from six classes and two different schools in Livorno, Italy: 63 children aged 9–10 years (*M*_age_ = 9.5 years, *SD* = 0.6), and 65 young students aged 14–16 years (*M*_age_ = 15.0 years, *SD* = 0.8). The pilot was followed by a spontaneous discussion in class, aimed at testing participants' understanding of the scenario, of the consequences of the actions presented in the scenario, and of the questions included in the questionnaire.

## Results

### Choice taken

Eighty-seven percent of the adolescents chose not to amputate, whereas only 27% of the children chose not to amputate, χ^2^(1,27) = 10.1, *p* = 0.001. 36% of the children (25% of which had chosen to amputate) and 41% of the adolescents (86% of which had chosen not to amputate) did not provide a reason for their choices. Most of the participants who provided a reason for their choices referred only to the anticipated outcomes of their choice. All participants who chose to amputate said that they would rather avoid suffering. Participants who decided not to amputate argued that they did not want to give up hope of once again being able to run, walk or do sport. Only few adolescents (N = 5) mentioned in their comments the information about the alternative outcomes presented in the scenario: “Even though the chances are low, they are there”

### Preference for autonomy

A univariate analysis of variance (ANOVA) indicated a main effect of condition on how much participants liked being assigned to the condition they were in, *F*(3,137) = 16.2, *p*<0.001, η^2^ = 0.3. A Bonferroni post hoc analysis confirmed that participants in Condition 1 (own informed choice) preferred to make the decision themselves more than participants in Conditions 2, 3, and 4 (parents' choice) preferred not to make the decision (*p*<0.001, Table 1). Post-hoc analyses (with Bonferroni correction) did not reveal any difference between Conditions 2, 3 and 4. Also, we found no differences between age groups or interaction effects.

An ANOVA with the choice made in Condition 1 as independent variable, confirmed that preference for autonomy was also not influenced by the choice made by participants in Condition 1, *p* = .329, nor by the age group, *p* = .933. The analysis did not reveal any interaction effect.

An ANOVA on how much participants wanted to change from their assigned condition to another condition indicated a main effect of condition, *F*(3,137) = 20.6, *p*<0.001, η^2^ = 0.30. Bonferroni post hoc analyses showed that participants assigned to Conditions 2, 3, and 4, where parents made the choice, were significantly more likely to want to change their condition than those in Condition 1 (own informed choice; *p*<0.001, Table 1). No significant differences emerged between conditions 2, 3 and 4 (all *p*s>0.05).

The analysis also showed a main effect of age, *F*(1,137) = 4.9, *p* = 0.029, η^2^ = 0.04: Children, overall, were less likely to desire to change condition than adolescents. Moreover, we found an Age × Condition interaction, *F*(3,137) = 3.37, *p* = 0.020, η^2^ = 0.07. Children in Condition 1 (own informed choice) were more willing to leave the decision to their parents than the adolescents in Condition 1, whereas adolescents in Conditions 2, 3, and 4 (where parents made the choice) were more interested than the children in being transferred to Condition 1. All post hoc analyses revealed no difference between Conditions 2, 3, and 4 (*p*>0.1): Providing information about the alternatives to parents as decision makers did not affect the preference for autonomy for either age group.

An ANOVA with the choice made in Condition 1 as independent variable, confirmed that willingness to change to a more autonomous condition was also not influenced by the choice made by participants in Condition 1, *p* = .557, nor by the age group, *p* = .456. The analysis did not reveal any interaction effect.

### Emotional response

We collapsed the participants' emotion ratings (nervous, upset, unhappy, concerned, guilty; Overall α = 0.77; Children α = 0.68; Adolescents α = 0.77) into one negative emotion score and conducted an ANOVA with condition and age as the independent variables. This analysis revealed the two main effects of condition, *F*(3,137) = 14.2, *p*<0.001, η^2^ = 0.25, and age, *F*(3,137) = 23.1, *p*<0.001, η^2^ = 0.15. As can be seen in Table 1, participants in Conditions 1 and 3 expressed significantly fewer negative emotions than participants in the other two conditions. All post hoc analyses revealed significant differences (*p*<0.001) between Condition 1 or 3 and Condition 2 or 4, whereas the emotional responses did not differ between Conditions 1 and 3 (*p* = 0.789) or between Conditions 2 and 4 (*p* = 0.731). Overall children reported fewer negative emotions than adolescents (see Table 1).

### Decision confidence

Regarding the participants' confidence that the best decision had been made, we found the two significant main effects of condition, *F*(3,137) = 14.5, *p*<0.001, η^2^ = 0.25, and age, *F*(3,137) = 7.2, *p* = 0.0008, η^2^ = 0.05. As displayed in Table 1, participants in Conditions 1 and 3 exhibited significantly higher confidence that the choice made was the best one compared to participants in Conditions 2 and 4. A Bonferroni post hoc analysis showed significant differences (*p*<0.001) between Condition 1 or 3 and Condition 2 or 4, and no differences between Conditions 1 and 3 (*p* = 0.805) or between Conditions 2 and 4 (*p* = 0.956). Children's confidence was overall lower than that of adolescents (see Table 1).

### Preference for autonomy or treatment choice?

The above analyses indicate children's and adolescents' emotional responses and decision confidence was affected by which choice condition they were assigned to (own choice, parents' choice), but also by the treatment choice (amputation, no amputation, including participants in Condition 1). We conducted two sets of hierarchical linear regression analyses to assess the influence of these two components (choice condition and treatment choice) on the dependent variables emotional response and decision confidence while controlling for (potential) age differences. Step 1 of the hierarchical linear regression analysis contained the independent variables choice condition (own vs. parents' choice), treatment choice (no amputation vs. amputation) and age group (children vs. adolescents). Step 2 additionally contained the interactions of Choice Condition × Age Group and Treatment Choice × Age Group.

As shown in Table 2, both age group and treatment choice significantly predicted emotional response, *F* (3, 135) = 20. 91, *p*<.001. Adolescents reported more negative emotional responses than children. Participants who decided to amputate reported significantly more negative emotional responses. Choice condition did not significantly predict emotional response. Regression model 2, which included the variables choice condition, treatment choice, and age group as well as the interaction terms of Choice Condition × Age group and Treatment Choice × Age Group did not lead to a significant change in *R* ^2^ compared to regression model 1, Δ*R* ^2^ = .02, Δ*F*(2, 133) = 2.01, *p* = .14 (Table 2). Therefore, the marginally significant interaction of Choice Condition × Age group was not further investigated.

**Table 2:** Results of Hierarchical Regression Analysis Predicting Emotional Response.

	Emotional response	
Independent variables	*β*	Δ*R^2^*, Δ*F*, *df*, *p*
Step 1		.32, 20.91, 3, .001
Choice condition	.13	
Treatment choice	.34**	
Age group	.41**	
Step 2		.02, 2.01, 2, .14
Choice condition	.14	
Treatment choice	.38**	
Age group	.96*	
Choice condition × Age group	−.69†	
Treatment choice × Age group	.12	

† *p*<.10 * p<.05, ** *p*<.01.

**Figure 1:**
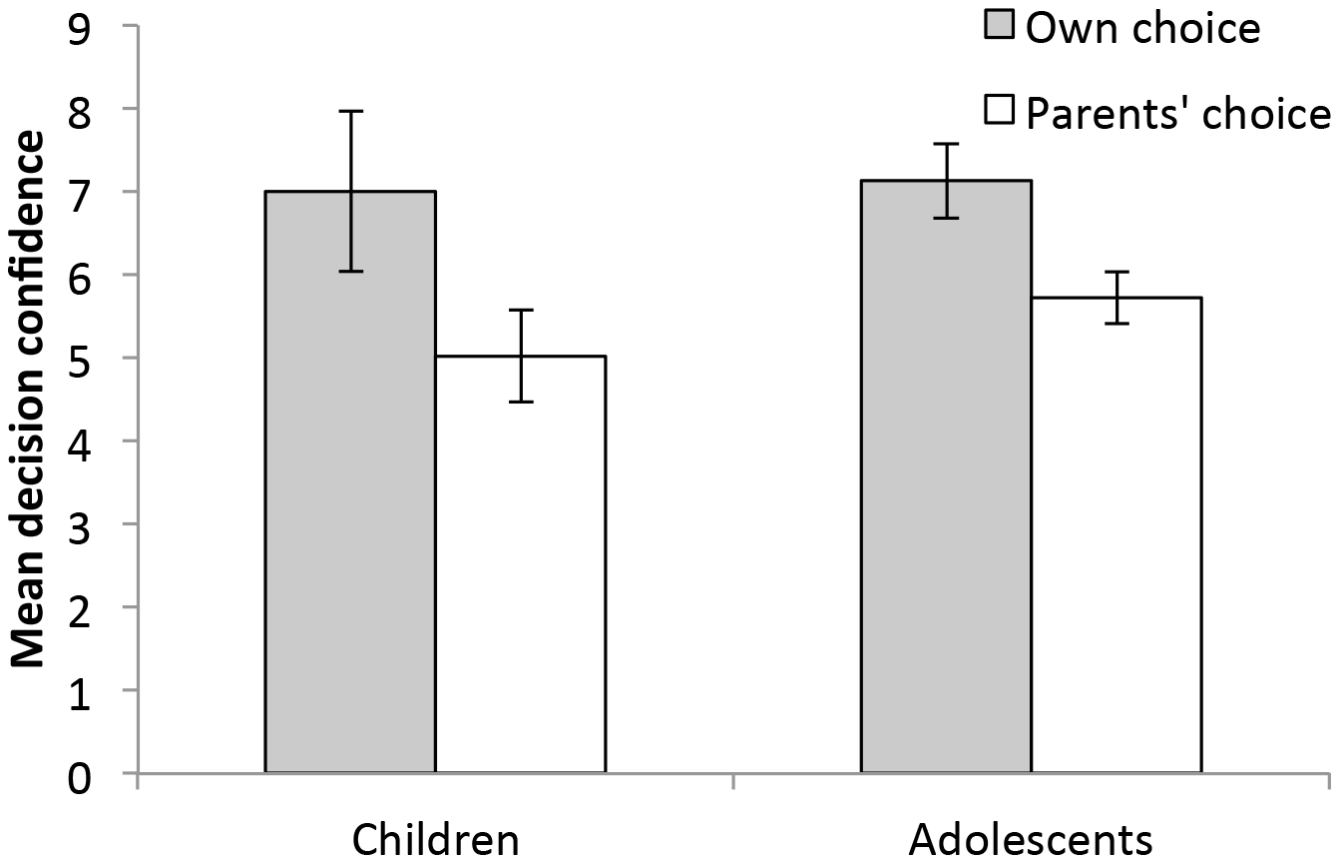
Mean decision confidence as a function of choice condition and age group. Error bars display standard errors.

Table 3 shows the results of the regression analyses for the dependent variable decision confidence. Regression model 1 revealed that both choice condition and treatment choice, but not age group, significantly predicted decision confidence, *F* (3, 134) = 13.13, *p*<.001. Participants who made their own choice were more confident about their decision. Furthermore, those who chose not to amputate showed higher decision confidence. Regression model 2, which additionally contained the interaction variables of Choice Condition × Age group and Treatment Choice × Age led to a significant change in *R* ^2^ compared to regression model 1, *ΔR* ^2^ = .04, *ΔF*(2, 132) = 3.66, *p* = .03. Table 3 shows that participants who made their own choice, those who chose not to amputate, and adolescents showed higher decision confidence. The interaction of Choice Condition × Age Group additionally predicted decision confidence. Subsequent regression analyses of the effect of choice condition on decision confidence within each age group showed that while participants in both age groups felt more confident in the own choice than parents' choice conditions, this difference was significant for adolescents, â = −.25, *t*(73) = 2.21, *p* = .03, but not for children, â = .23, *t*(61) = 1.82, *p* = .10 (see Figure 1).

**Table 3:** Results of Hierarchical Regression Analysis Predicting Decision Confidence.

	Decision Confidence	
Independent variables	*β*	Δ*R^2^*, Δ*F*, *df*, *p*
Step 1		.23, 13.13, 3, .001
Choice condition	−.20*	
Treatment choice	−.40**	
Age group	.14	
Step 2		.04, 3.65, 2, .03
Choice condition	−.25**	
Treatment choice	−.48**	
Age group	.97*	
Choice condition × Age group	−.92*	
Treatment choice × Age group	.27	

* p<.05, ** *p*<.01.

## Discussion

The United Nations Convention on the Rights of the Child, the US Supreme Court, and, most importantly, the medical establishment, at least in the United States, have all come to recognize the importance of giving minors a say in making medical decisions. So far, researchers have tended to focus on the relationship between minors' cognitive abilities and decision competence [5], [2], [4]. With few exceptions (see[16]), what has been missing is insight into whether children and adolescents want to be involved in the process of making decisions about their medical treatment even when those decisions are difficult and might be emotionally taxing for them.

Our data clearly indicate that children and adolescents want to be involved in the decision process, even when the outcome involves serious negative consequences. Participants preferred making the decision themselves rather than having an authority figure (a parent) decide for them. Desire for autonomy was independent of the decision made by parents (i.e., amputate in Condition 2 vs. not amputate in Condition 3) and of the decision made by participants in Condition 1 (i.e., whether to amputate or not). As hypothesized (Hypothesis 1), this willingness to make autonomous decisions and not to let parents make the choice was stronger for adolescents than children. Our findings, thus, are nicely aligned with results of previous developmental research showing adolescents' greater desire for autonomous decision making in more everyday contexts with less difficult outcomes (e.g.,[9], [10]). Adolescents might feel that they are grown up and as such deserve to be independent and are entitled to decide about their own medical treatment.

In Condition 1, most of the adolescents (87%) chose not to amputate, whereas only 27% of the children chose not to amputate. This was an unexpected result. Children consistently reported to be worried about feeling pain for their entire life if they do not amputate. This was the only other alternative to amputation mentioned by the doctor in the given scenario. Because the doctor is an expert adult, it is not too surprising that children believed that the given alternatives were the only two available and decided to avoid the possibility of future pain and amputate. They might even have perceived that the doctor was indirectly suggesting that it would have been better to amputate, because he presented the other alternative as very unattractive. Indeed, two children explicitly mentioned that “this is what the doctor would do”. Adolescents, in contrast, reported that they “did not want to give up” and to “believe there was still hope of saving the leg without necessarily having to suffer in the future”, even though this possibility was not mentioned by the doctor in the scenario.

This result might relate to adolescents' well-documented illusion of invincibility [17]. Invincibility is a typical phase of social and cognitive development of adolescence that peaks in early adolescence and is dominated by egocentric thinking, a side effect of the teen's search for identity. Teens believe that they are the focus of everyone's attention and are constantly being evaluated by others. This belief further engenders feelings of uniqueness, as teens perceive their feelings and experiences as exceptional and not subject to the laws governing others' lives, and promote the illusion of being special and invulnerable to the consequences of dangerous or risky behavior [18], [19]. Such illusion and feeling of uniqueness might help explaining why adolescents, ignoring the options given by the doctor, thought there was still a chance for them to save their legs without having to suffer pain forever.

We know that adolescents are very accurate and predictive when they make probability judgments for a number of significant life events, except for judging the probability of dying prematurely [20], [21]. What about children's and adolescents' ability to forecast their emotional reactions to difficult choices? Even though there has been a growing interest in adults' ability to forecast their emotional responses to various health decisions and conditions [22], [23], to our knowledge, this line of investigation has not been applied to minors (see [24]). Botti et al. [6] proposed that personal responsibility was associated with greater negative emotional responses (Hypothesis 2). However, we found that participants in Condition 1 (own informed choice) reported similar negative emotions to those of participants in Condition 3 (informed parents' choice to continue treatment), and lower negative emotional responses than participants in Conditions 2 and 4 (informed parents' choice to amputate; uninformed parents' choice to amputate). In this sense, it is evident that the choice condition alone is not enough to predict participants' emotional responses, but the decision outcome (amputate vs. not amputate) has to be considered as well. Indeed, participants reported lower negative emotional responses when the decision choice was “no amputation”. Future research might systematically vary the seriousness of the decision outcome and investigate its effect on emotional responses.

Treatment choice (amputate vs. not amputate) also affected decision confidence, and our results support both Hypotheses 3a and 3b: Participants reported higher confidence that the right decision has been made when they themselves (versus the parents) made the decision. Furthermore, those who chose not to amputate expressed higher decision confidence.

Moreover, children's decision confidence was overall lower than that of adolescents, and they also reported fewer negative emotions than adolescents. A possible interpretation of these results is that children are less able than adolescents to project how they would feel, that is, to form a counterfactual scenario of how it would feel to have lost a leg or live with pain (see[25], [26]).

In contrast to Botti et al.'s findings [6], we also found that not providing information about the alternatives at stake (in Condition 4 compared to Condition 2) did not affect participants' responses (see Hypothesis 4). These results might be due to children's and adolescents' inability to conceptualize and utilize the information provided. This result reinforces the need to design health and risk communications in a transparent and easy-to-understand way for patients of all ages [27]–[29].

Our study is not without limitations. First, our sample is one of convenience and the study was conducted at school rather than in a clinic or in a hospital. Second, the scenarios presented to children were hypothetical by nature and only focused on a single health related problem. It is unclear whether our results are robust enough to generalize to other health issues such as diabetes or cancer. While future studies should examine clinical samples, our novel results, nonetheless, highlight the need to further explore children's and adolescents' desire to be actively involved in their health decision making.

In conclusion, our results suggest that age and cognitive competence are not the only factors that should be taken into account when considering whether minors *deserve* a voice in medical decision making. Children and adolescents want to be involved in medical decisions, even when the choice is a difficult one. A future direction would be to investigate how medical decisions are and should be *negotiated* within families, for example, to minimize the negative emotional impact the choice and the choice outcomes have on all family members. This line of research would tap not only into the literature on shared decision making about health [30]–[32], but also into the more recent studies reporting systematic differences between the treatment choice one recommends for another person vs. makes for oneself (see [23], [33]). How can we better involve minors and their families in the process of making medical decisions?
